# African Swine Fever Virus p72 Genotype IX in Domestic Pigs, Congo, 2009

**DOI:** 10.3201/eid1708.101877

**Published:** 2011-08

**Authors:** Carmina Gallardo, Raquel Anchuelo, Virginia Pelayo, Frédéric Poudevigne, Tati Leon, Jacques Nzoussi, Richard Bishop, Covadonga Pérez, Alejandro Soler, Raquel Nieto, Hilario Martín, Marisa Arias

**Affiliations:** Author affiliations: Centro de Investigación en Sanidad Animal, Valdeolmos, Madrid, Spain (C. Gallardo, V. Pelayo, C. Pérez, A. Soler, R. Nieto, H. Martín, M. Arias);; International Livestock Research Institute, Kabete, Nairobi, Kenya (R. Anchuelo, R. Bishop);; United Nations Food and Agriculture Organization, Bamako, Mali (F. Poudevigne);; Conseiller à l'Elevage du Ministre de l'Agriculture et de l'Elevage, Brazzaville, Republic of the Congo (T. Leon, J. Nzoussi)

**Keywords:** African swine fever virus, p72, p54, central variable region, genotyping, viruses, Congo, swine, arboviruses, dispatch

## Abstract

African swine fever virus p72 genotype IX, associated with outbreaks in eastern Africa, is cocirculating in the Republic of the Congo with West African genotype I. Data suggest that viruses from eastern Africa are moving into western Africa, increasing the threat of outbreaks caused by novel viruses in this region.

African swine fever (ASF) is a serious disease of domestic pigs caused by a DNA arbovirus (African swine fever virus [ASFV]) belonging to the family *Asfaviridae* (*1*). Its highly contagious nature and ability to spread over long distances make it 1 of the most feared diseases of pigs; it causes devastating effects on pig production as manifested in the Caucasus since its introduction from southeastern Africa during 2007 (*2*). Considerable spread of ASF has been reported in western Africa during the past 20 years, and, except for in Côte d’Ivoire, the disease remains endemic (*3*). Because discernible ASFV serotypes are lacking, the field strains are grouped genetically by using sequencing of the C-terminus of the p72 protein, which discriminates 22 genotypes (*4**,**5*). Genotype I is historically associated with outbreaks in western Africa, whereas viruses from southern and eastern Africa have higher heterogeneity, with all 22 known genotypes having been recorded within the region (*5**–**7*).

The Republic of the Congo, located in western-central sub-Saharan Africa, shares borders with the Cabinda enclave of Angola, the Democratic Republic of the Congo, Central African Republic, Cameroon, and Gabon. The last ASF outbreaks in Congo were reported to the World Organization for Animal Health (OIE) during 2003. Since then, the disease has been officially declared endemic but without quantitative data. Sampling and characterization of currently circulating field strains from this region of western-central Africa are needed to fully understand virus spread and maintenance. Such data will have implications for regional control in western Africa.

## The Study

During August 2009, a United Nations Food and Agriculture Organization mission was undertaken with local counterparts in Congo to support development of an action plan to control ASF. A key factor in selecting the sites sampled was inclusion of the main pig-producing, marketing, and consuming areas, with a particular focus where suspected ASF outbreaks had been recently reported. From the survey conducted, 86 samples comprising serum (35 samples), whole blood (44 samples), and tissues (7 samples) were collected from 80 domestic pigs in the departments of Brazzaville, Pointe Noire, Kouilou, Bouenza, Niari, and Cuvette (Figure). These departments contain 80%–90% of the country’s pig population. Samples also were collected from Pool in southeastern Congo, where a recent hemorrhagic disease outbreak, characterized by case-fatality rates of ≈80%, had been reported. Clinical material was sent to the European Union Reference Laboratory for African Swine Fever (Centro de Investigación en Sanidad Animal, Madrid, Spain) for confirmatory diagnosis and characterization of the ASFV strain(s) responsible for the outbreak(s).

**Figure:**
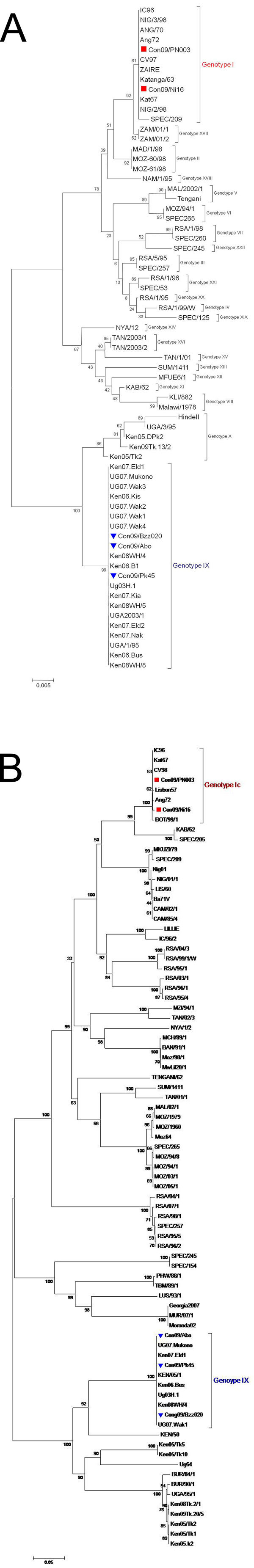
Republic of the Congo, showing location of the African swine fever virus isolates from eastern (blue) and western (red) Congo selected for genotyping.

Specific ASF antibodies were detected by using the OIE-prescribed assays (*8*) in 7 of 35 serum samples analyzed. All positive serum samples were from animals that had survived the ASF outbreaks in Bouenza, Niari, and Pointe Noire during 2008. For ASFV genome detection, OIE-prescribed PCRs (*8*) were performed on DNA extracted from 28 serum samples, 44 blood samples, and 7 tissue homogenates. A positive result was obtained in 17 (21%) of samples analyzed, indicating ASF in all the departments where the survey was conducted except for Kouilou.

**Figure A1:**
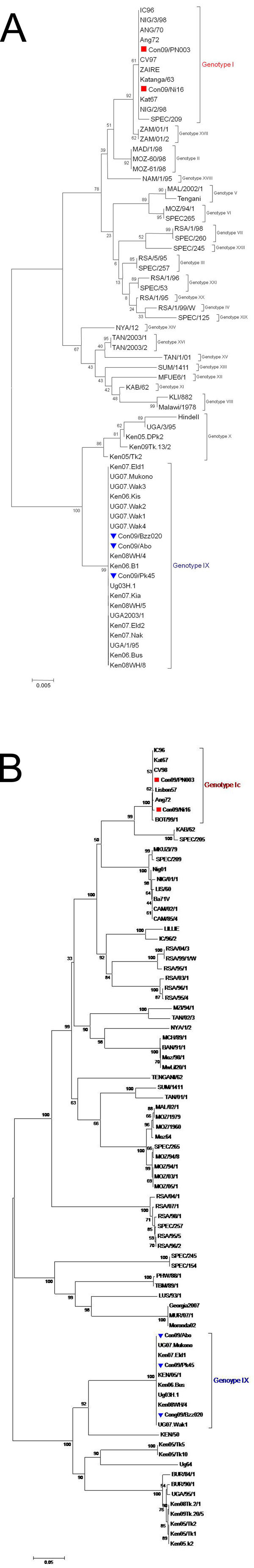
Minimum-evolution (ME) phylogenetic trees of 5 isolates from Republic of the Congo, 2009, based on C-terminal end of the p72 protein relative to the 22 p72 genotypes (labeled I–XXII) involved 6-nt sequences (A) and full length *p54* gene sequences among 90 African swine fever virus (ASFV) isolates (B). The trees were inferred by using the ME method after initial application of a neighbor-joining algorithm. The percentage of replicate trees in which the associated taxa clustered by bootstrap analysis (1,000 replicates) is shown adjacent to the nodes. The robustness of the ME trees was tested by using the close-neighbor interchange algorithm at a search level of 1. ASFV isolates from western Congo are indicated by red squares, ASFV isolates from eastern Congo by blue triangles.

Subsequently, 5 hemadsorbent Congo ASFVs (Table) were isolated in porcine peripheral blood macrophages (*8*). Viral DNA was extracted, and 3 different sets of primers were used for ASFV genotyping. A region of 478 bp at the C-terminal end of the p72 protein and the full-length sequence of *p54* gene were amplified by using primers p72U/D and 89/722, respectively (*6*). We compared the sequence from each of the p72 and p54 amplicons with homologous sequences representative of each previously described p72 and p54 genotype (*5**,**6*). A rooted minimum-evolution tree was constructed by using MEGA4.0 software and the p*-*distance nucleotide substitution model (*9*). Two different ASFV phylogenetic profiles were found in western and southeastern Congo. The phylogeny inferred for the ASFV isolates from eastern Congo (Con09/Pk45, Con09/Bzz020, Con09/Abo) placed them within p72 and p54 genotype IX with viruses collected from recent outbreaks in Kenya and Uganda during 2006–2007 (*6**,**7*). By contrast, ASFV isolates from western Congo (Con09/Ni16, Con09/PN003) clustered in p72 genotype I and p54 genotype Ic (*6*) comprising historical isolates from western Africa, such as Ang72 and Kat67, and the first ASFV isolated from Europe, the Portuguese Lisbon57 isolate (Figure A1).

**Table:** African swine fever virus isolates selected for genotyping from domestic pigs after virus isolation for which nucleotide sequence was determined at 3 loci, Republic of the Congo, 2009*

Isolate	Sampling location				p72			p54			Central variable region	
	Locality	Dept	Province		Geno	GenBank		Geno	GenBank		Subgroup	GenBank
Con09/PN003	Tchimbamba	Ponte Noire	Southwestern		I	HQ645947		Ic	HQ645949		XIII	HQ645957
Con09/Ni16	Kikassa	Niari	Western		I	HQ645943		Ic	HQ645948		Related XXVI	HQ645953
Con09/Pk45	Ferme Champenoise Dimi, Igné	Pool	Southeastern		IX	HQ645944		IX	HQ645952		XXIV	HQ645954
Con09/Bzz020	Ferme Champenoise Dimi, Igné	Pool	Southeastern		IX	HQ645945		IX	HQ645950		XXIV	HQ645955
Con09/Abo	Ferme Ibovi	Abo, Cuvette	Eastern		IX	HQ645946		IX	HQ645951		XXIV	HQ645956

*Dept, department; geno, genotype; GenBank, GenBank accession number.

To provide higher resolution, the tetrameric tandem amino acid repeat sequences within central variable region (CVR) of the *B602L* gene were analyzed (*5**–**7**,**10*). Primers CVR1 (5′-ACTTTGAAACAGGAAAC(AT)AATGATG-3′) and CVR2 (5′-ATATTTTGTAATATGTGGGCTGCTG-3′) were designed in this study selected from complete genome sequences of ASFV strains available in GenBank.

Under the same thermal cycling conditions used for full *p54* gene amplification, amplicons of 600–650 bp were generated from the isolates from eastern Congo, whereas the estimated size of the amplicons from western Congo was 500 bp (data not shown). Analysis of the tandem amino acid repeat sequences within the CVR demonstrated 3 different variants cocirculating in the country, 2 within the p72 type I virus genotype. The type of CVR sequence (AABNABTDBNAAAA) identified in the isolate from Pointe Noire (Con09/PN003) was identical to CVR subgroup XIII (*11*) that contains early isolates from Angola (Ang70, Ang72) and Portugal (Lis57). However, the Con09/Ni16, also classified within p72 and p54 genotypes I and Ic, showed a unique CVR sequence (AAAAAAAAAF) not previously described but most similar to viruses from Burundi and Kenya in CVR subgroup XXVI (*7**,**11*). As for p72 and p54 genotyping, isolates from eastern Congo were related to CVR subgroup XXIV, which contains isolates obtained during the 2006 and 2007 outbreaks in Uganda and Kenya (*6**,**7*).

## Conclusions

We confirmed ASF in 5 of the 6 departments in which surveillance was conducted during August 2009 in Congo. Genotyping of 5 ASFV isolates from Congo resulted in identification of genetically distinct viruses circulating simultaneously in the country. In eastern districts of Congo, viruses were most genetically similar to those recovered from the outbreaks in Kenya and Uganda during 2006 and 2007. Therefore, genotype IX, associated with ASF outbreaks in eastern Africa during the previous 10 years, also occurs in western Africa. By contrast, viruses in western Congo were similar to genotype I viruses that historically circulated in western Africa. Analyses of the CVR identified 2 discrete variants within genotype I. The Pointe Noire ASFV was identical to viruses isolated in neighboring Angola during the 1970s, indicating a prolonged persistence of this virus type in the region. By contrast, the ASFV isolated from Niari was related to variants circulating during the 1980s in Burundi. These results are consistent with recent transfer and dissemination of ASFV genotypes from eastern to western Africa. Broadening of our initial findings requires extending analyses to western-central Africa, particularly to countries such as Cameroon and the Democratic Republic of the Congo, and providing more in-depth analysis in Congo.

The apparent recent movement of a virus genotype previously associated with virulent ASF in eastern Africa to western Africa, where the viruses have hitherto always been classified in p72 genotype I, indicates the possibility of future outbreaks of disease caused by novel viruses in western Africa. The reality of this scenario recently was demonstrated in the Caucasus region. Our study confirms the continuing spread of ASFV.
